# Supplementary material for: Phagocytosis of SARS-CoV-2 nucleocapsid- and RNA-containing immune complexes drives inflammatory cytokine production and endothelial dysfunction
Source: PLoS Pathog. 2026 Oct 29;22(10):e1014664. doi: 10.1371/journal.ppat.1014664 (PMC13739219; doi:10.1371/journal.ppat.1014664)
Supplement: Supplementary file 2 [file ppat.1014664.s002.pdf]

S2 Fig

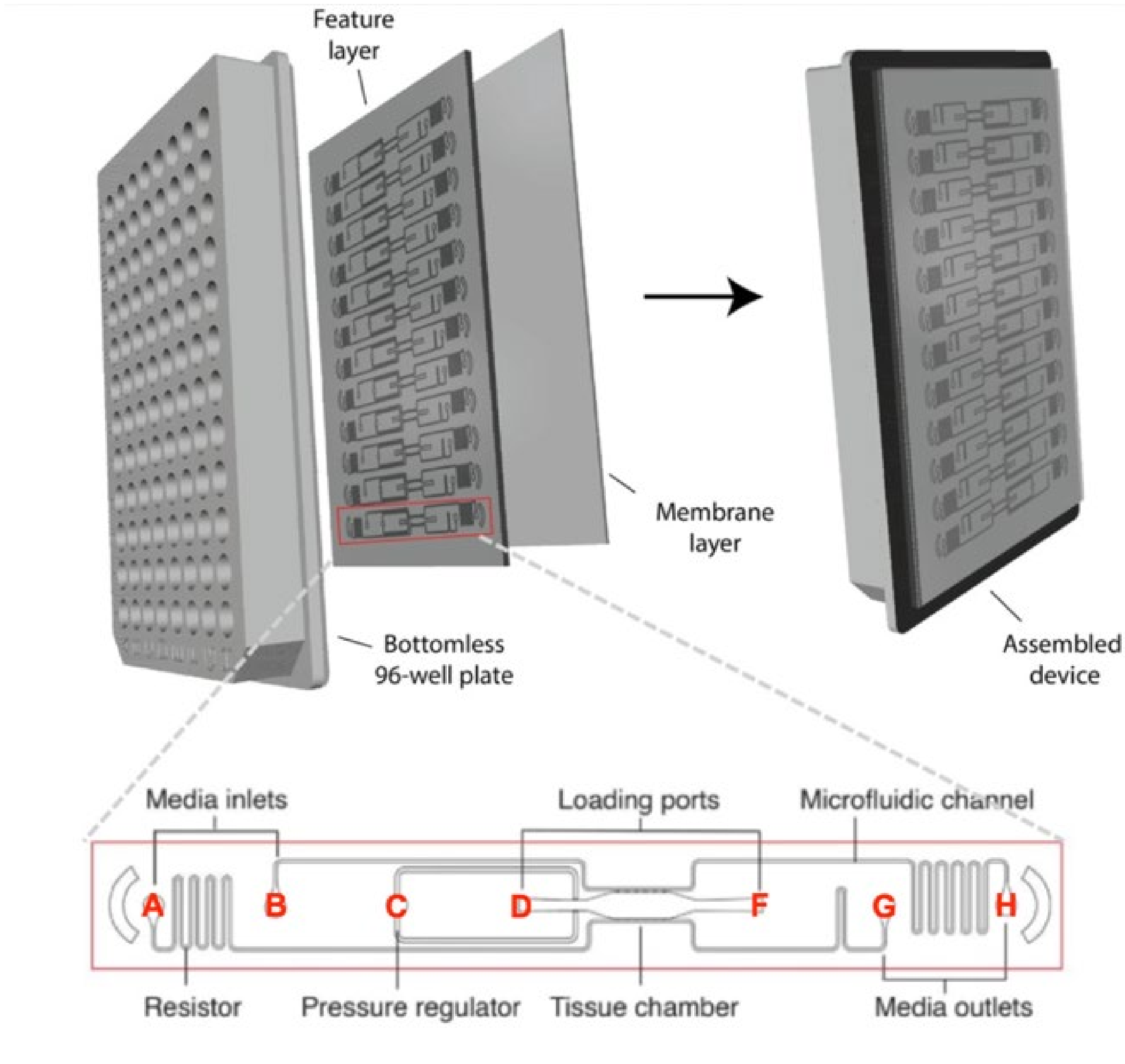

Feature layer
Membrane layer
Bottomless 96-well plate
Assembled device
Media inlets
Loading ports
Microfluidic channel
A
B
C
D
F
G
H
Resistor
Pressure regulator
Tissue chamber
Media outlets
