# Supplementary material for: Phagocytosis of SARS-CoV-2 nucleocapsid- and RNA-containing immune complexes drives inflammatory cytokine production and endothelial dysfunction
Source: PLoS Pathog. 2026 Oct 29;22(10):e1014664. doi: 10.1371/journal.ppat.1014664 (PMC13739219; doi:10.1371/journal.ppat.1014664)
Supplement: Supplementary file 3 [file ppat.1014664.s003.pdf]

S3 Fig

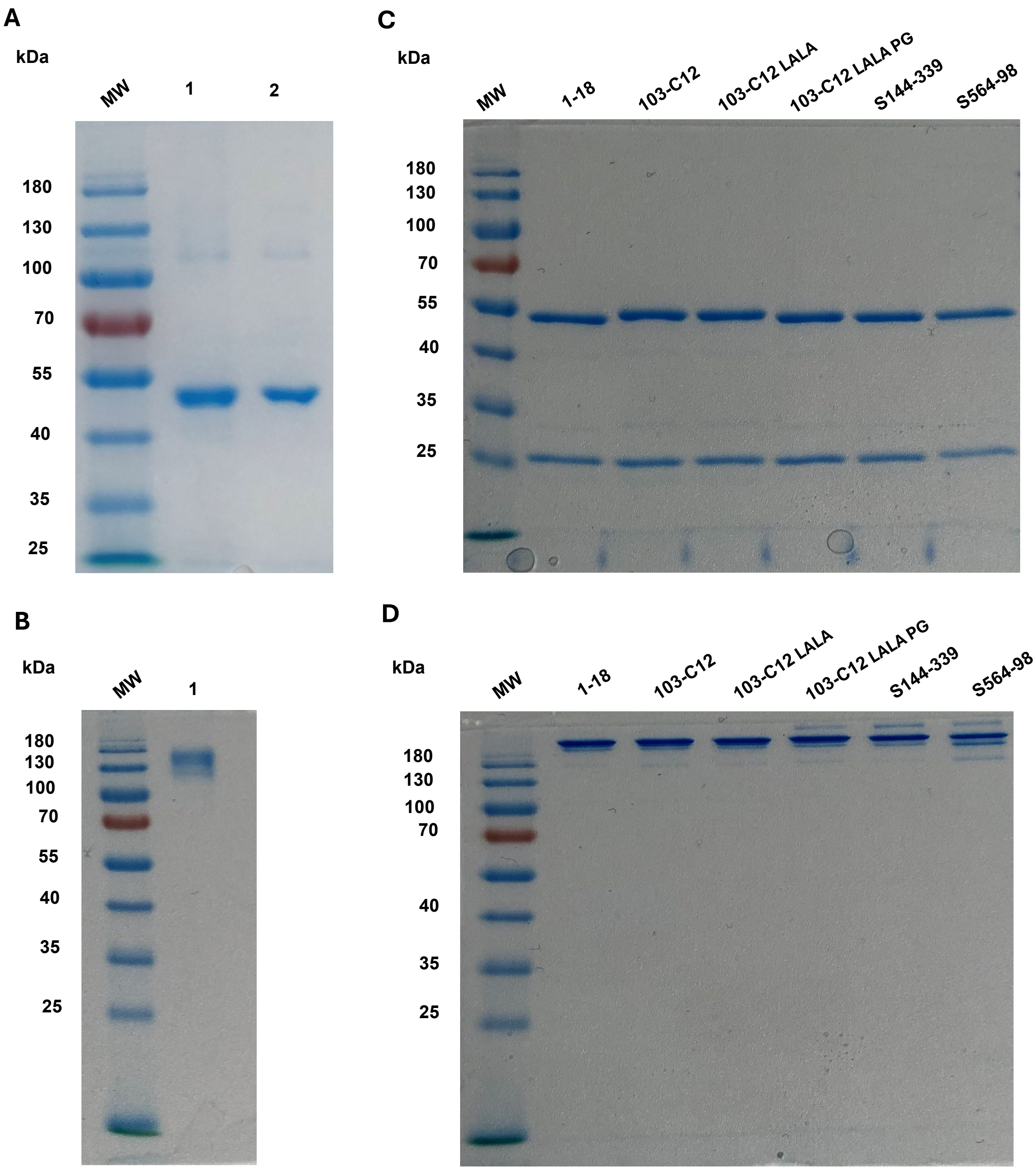
A
kDa
MW
1
2
180
130
100
70
55
40
35
25
C
kDa
MW
1-18
103-C12
103-C12 LALA
103-C12 LALA PG
S144-339
S564-98
180
130
100
70
55
40
35
25
B
kDa
MW
1
180
130
100
70
55
40
35
25
D
kDa
MW
1-18
103-C12
103-C12 LALA
103-C12 LALA PG
S144-339
S564-98
180
130
100
70
40
35
25
