# Supplementary material for: Phagocytosis of SARS-CoV-2 nucleocapsid- and RNA-containing immune complexes drives inflammatory cytokine production and endothelial dysfunction
Source: PLoS Pathog. 2026 Oct 29;22(10):e1014664. doi: 10.1371/journal.ppat.1014664 (PMC13739219; doi:10.1371/journal.ppat.1014664)
Supplement: Supplementary file 4 [file ppat.1014664.s004.pdf]

S4 Fig

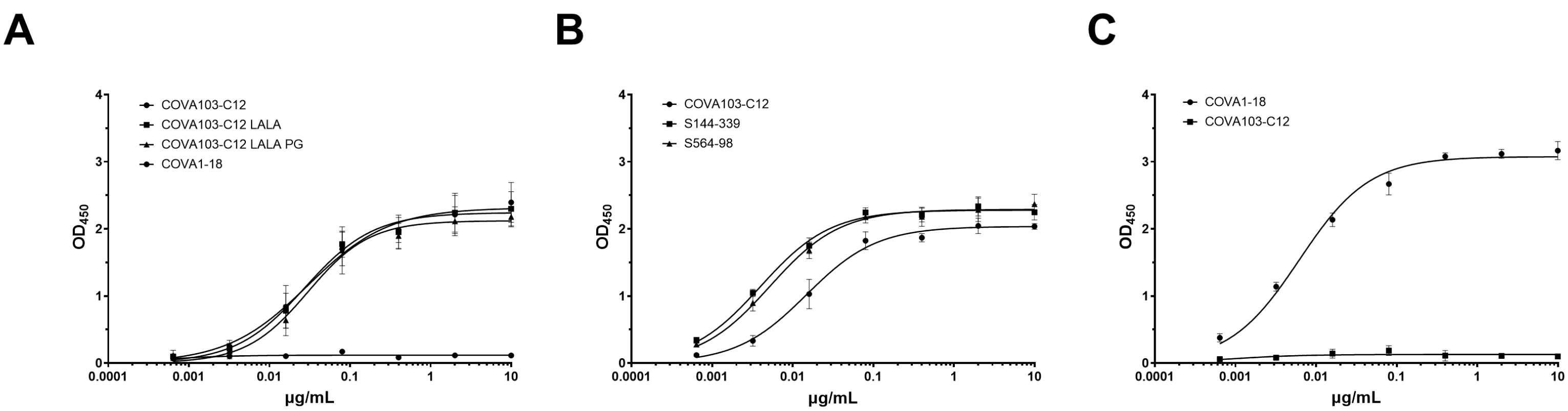

A
COVA103-C12
COVA103-C12 LALA
COVA103-C12 LALA PG
COVA1-18
OD450
µg/mL
B
COVA103-C12
S144-339
S564-98
OD450
µg/mL
C
COVA1-18
COVA103-C12
OD450
µg/mL
