# Supplementary material for: Phagocytosis of SARS-CoV-2 nucleocapsid- and RNA-containing immune complexes drives inflammatory cytokine production and endothelial dysfunction
Source: PLoS Pathog. 2026 Oct 29;22(10):e1014664. doi: 10.1371/journal.ppat.1014664 (PMC13739219; doi:10.1371/journal.ppat.1014664)
Supplement: Supplementary file 5 [file ppat.1014664.s005.pdf]

S5 Fig

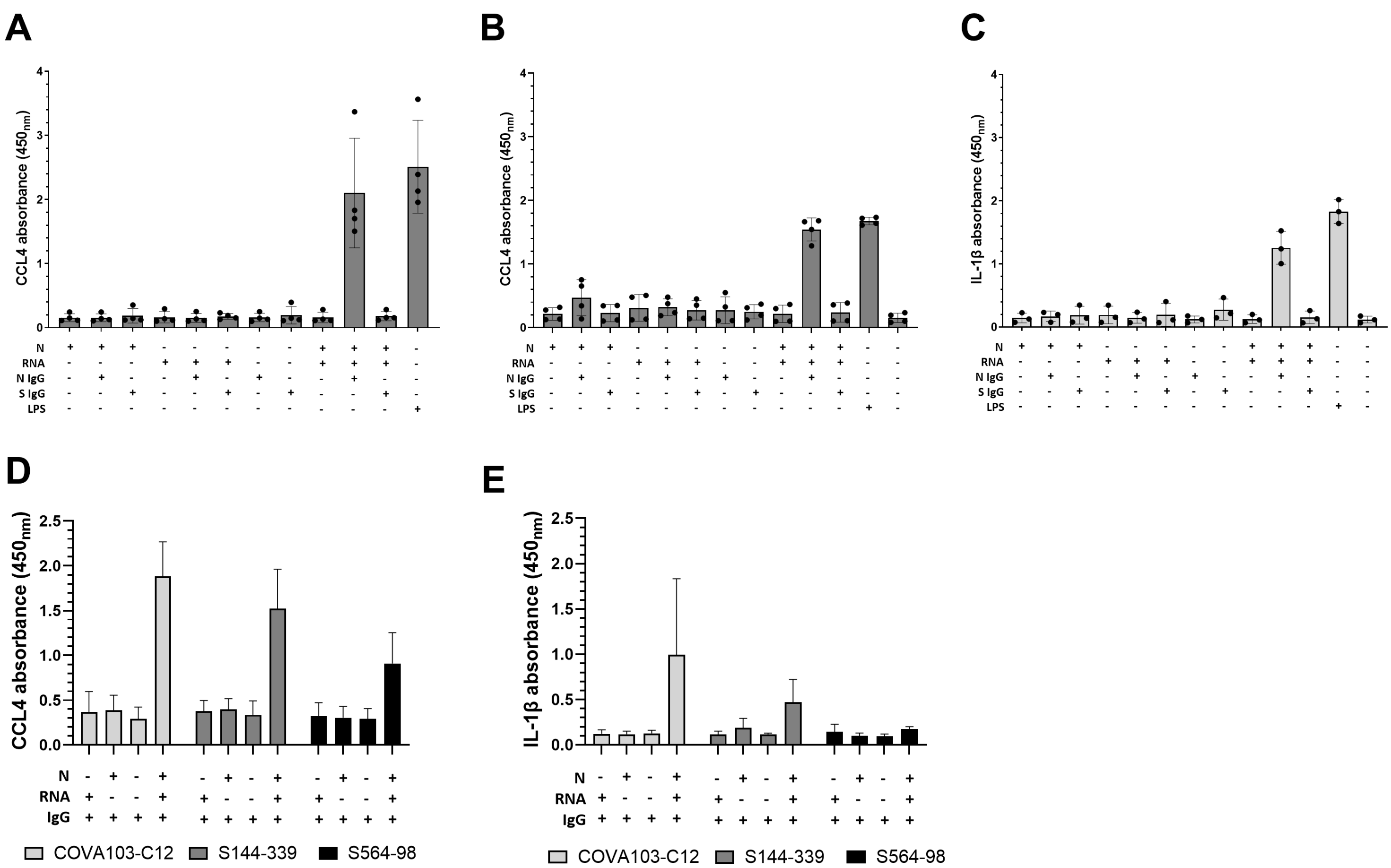

A
B
C
D
E
CCL4 absorbance (450nm)
IL-1β absorbance (450nm)
N
RNA
N IgG
S IgG
LPS
IgG
COVA103-C12
S144-339
S564-98
