# Supplementary material for: Phagocytosis of SARS-CoV-2 nucleocapsid- and RNA-containing immune complexes drives inflammatory cytokine production and endothelial dysfunction
Source: PLoS Pathog. 2026 Oct 29;22(10):e1014664. doi: 10.1371/journal.ppat.1014664 (PMC13739219; doi:10.1371/journal.ppat.1014664)
Supplement: Supplementary file 6 [file ppat.1014664.s006.pdf]

S6 Fig

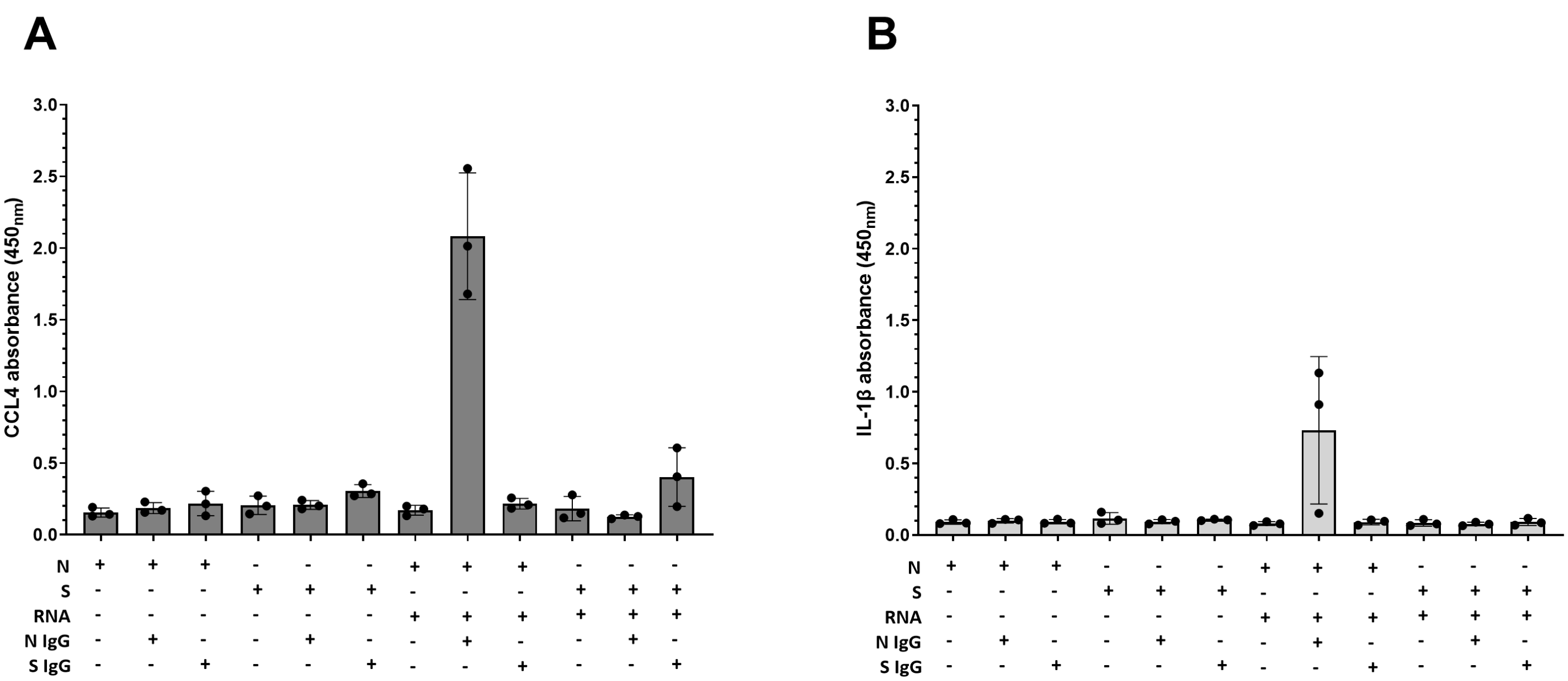
A
B
CCL4 absorbance (450nm)
IL-1β absorbance (450nm)
3.0
2.5
2.0
1.5
1.0
0.5
0.0
N
S
RNA
N IgG
S IgG
