# Supplementary material for: Phagocytosis of SARS-CoV-2 nucleocapsid- and RNA-containing immune complexes drives inflammatory cytokine production and endothelial dysfunction
Source: PLoS Pathog. 2026 Oct 29;22(10):e1014664. doi: 10.1371/journal.ppat.1014664 (PMC13739219; doi:10.1371/journal.ppat.1014664)
Supplement: Supplementary file 7 [file ppat.1014664.s007.pdf]

S7 Fig

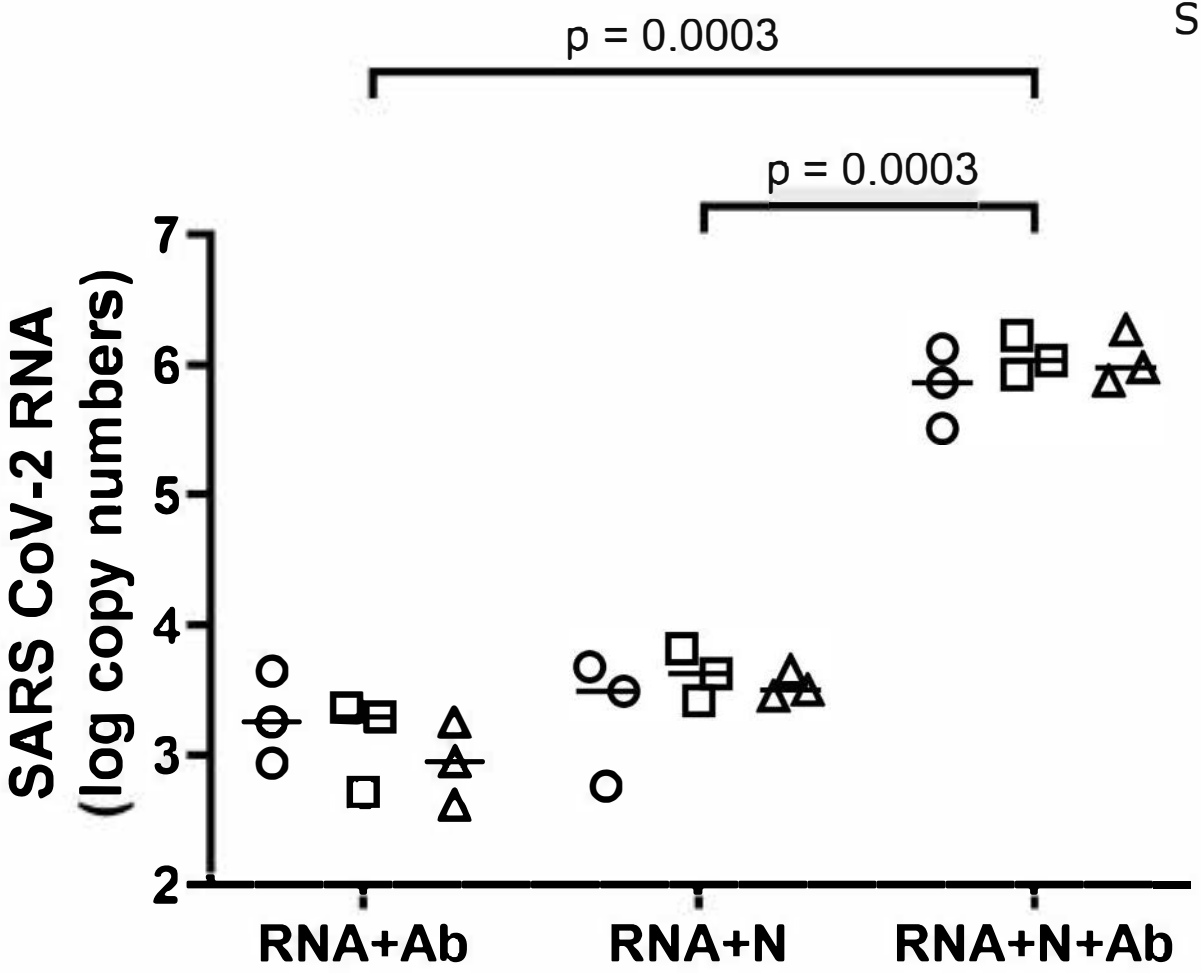
p = 0.0003
p = 0.0003
SARS CoV-2 RNA
(log copy numbers)
7
6
5
4
3
2
RNA+Ab
RNA+N
RNA+N+Ab
