# Supplementary material for: Phagocytosis of SARS-CoV-2 nucleocapsid- and RNA-containing immune complexes drives inflammatory cytokine production and endothelial dysfunction
Source: PLoS Pathog. 2026 Oct 29;22(10):e1014664. doi: 10.1371/journal.ppat.1014664 (PMC13739219; doi:10.1371/journal.ppat.1014664)
Supplement: Supplementary file 8 [file ppat.1014664.s008.pdf]

S8 Fig

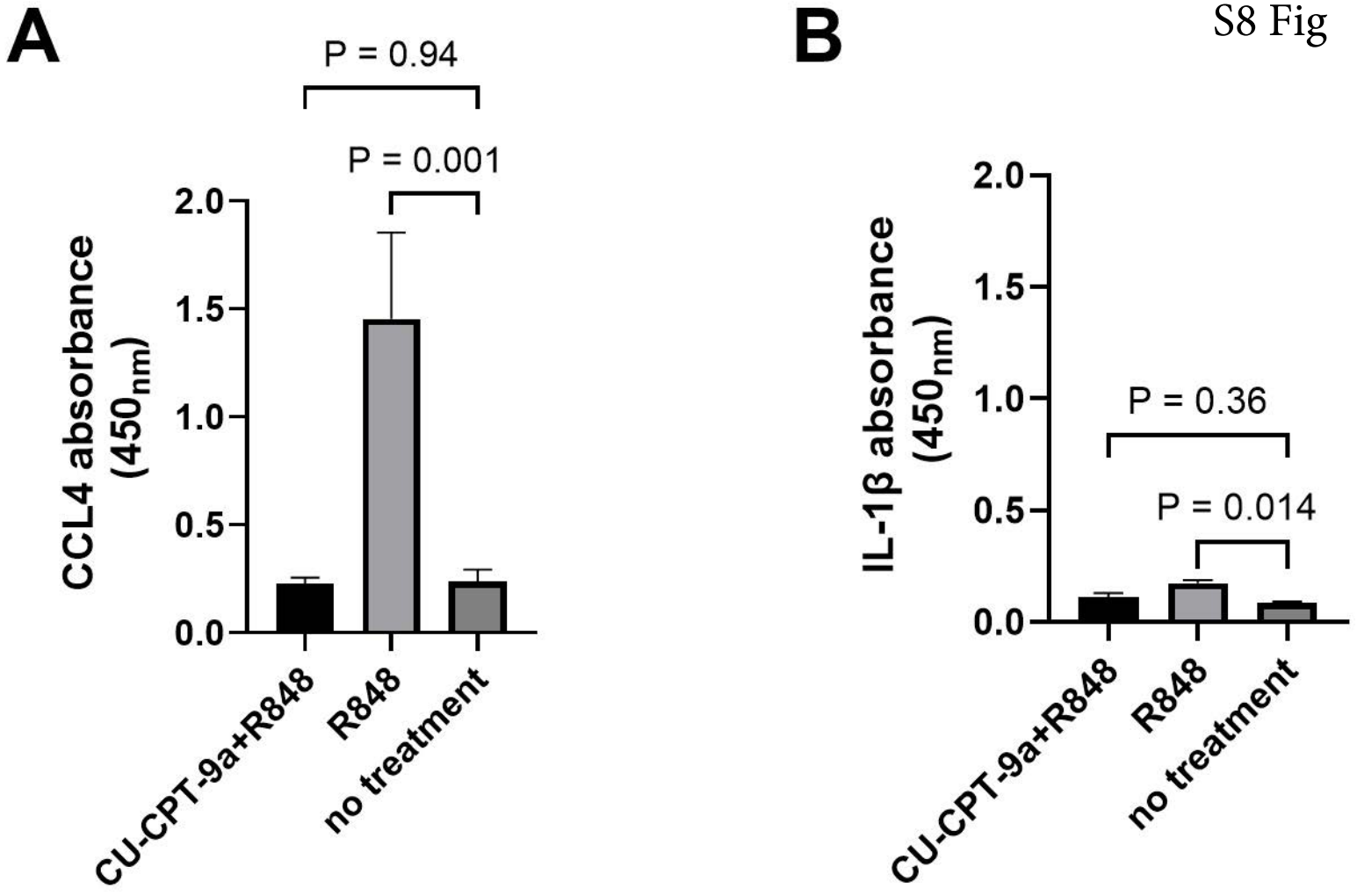
A
P = 0.94
P = 0.001
CCL4 absorbance (450nm)
2.0
1.5
1.0
0.5
0.0
CU-CPT-9a+R848
R848
no treatment
B
IL-1β absorbance (450nm)
2.0
1.5
1.0
0.5
0.0
P = 0.36
P = 0.014
CU-CPT-9a+R848
R848
no treatment
