# Supplementary material for: Phagocytosis of SARS-CoV-2 nucleocapsid- and RNA-containing immune complexes drives inflammatory cytokine production and endothelial dysfunction
Source: PLoS Pathog. 2026 Oct 29;22(10):e1014664. doi: 10.1371/journal.ppat.1014664 (PMC13739219; doi:10.1371/journal.ppat.1014664)
Supplement: Supplementary file 9 [file ppat.1014664.s009.pdf]

Uncropped gel images captured by iPhone

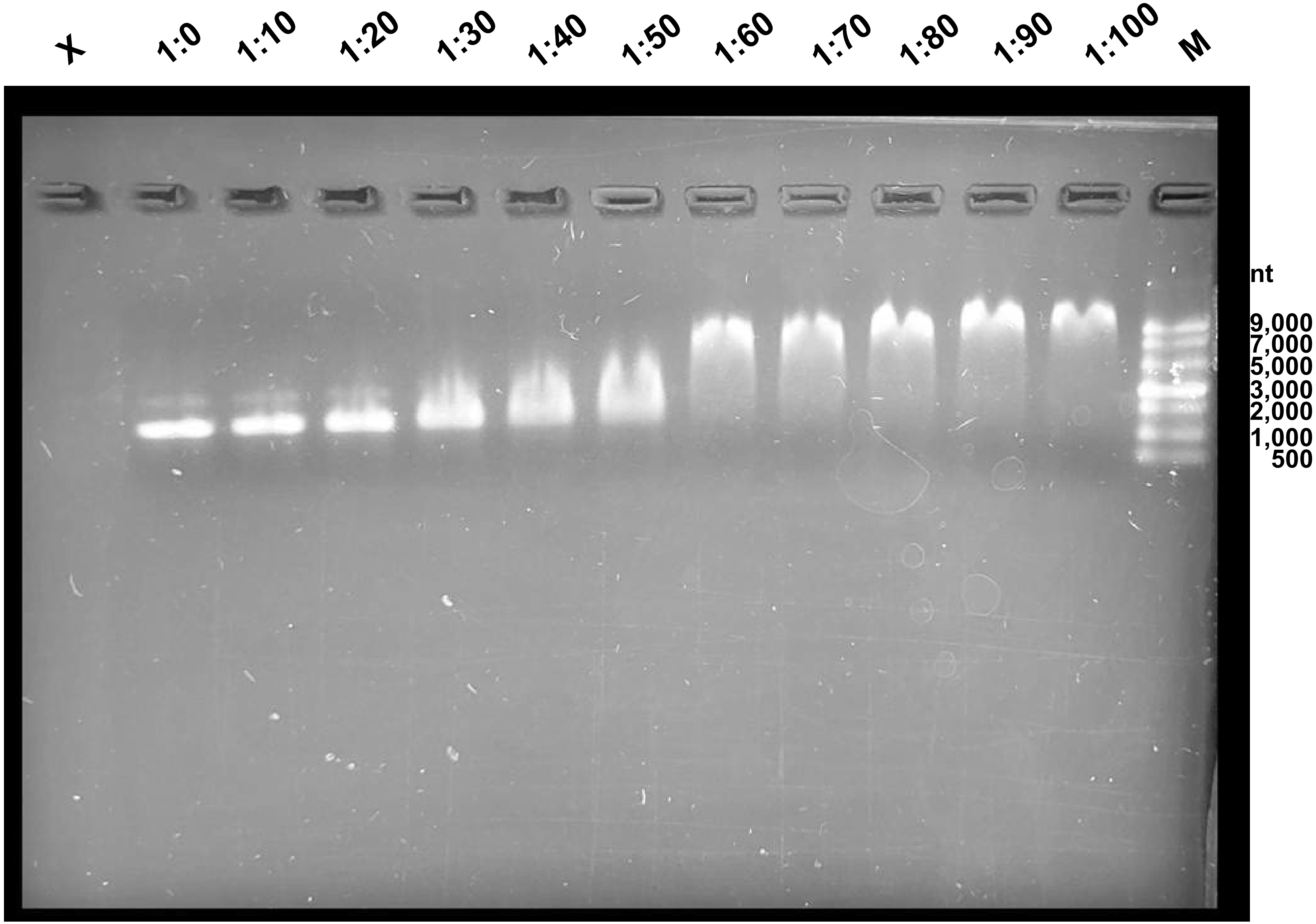

Figure 1 (A) In vitro transcribed SARS-CoV-2 RNA (from the N-coding region) was incubated with increasing concentrations of purified N to achieve the indicated molar ratios (RNA:N = 1:0 to 1:100). The resulting ribonucleoprotein complexes were resolved on a 1% non-denaturing agarose gel. Lane M contains an ssRNA ladder (sizes in nucleotides, nt, are indicated on the right) used to estimate the size and integrity of the input RNA (~1200 nt). The progressive reduction in the intensity of the free RNA band and the appearance of a shifted, less mobile band (or smear at lower concentrations) demonstrates a dose-dependent binding interaction. Complete sequestration of the free RNA band is observed at a 1:60 molar ratio and higher.

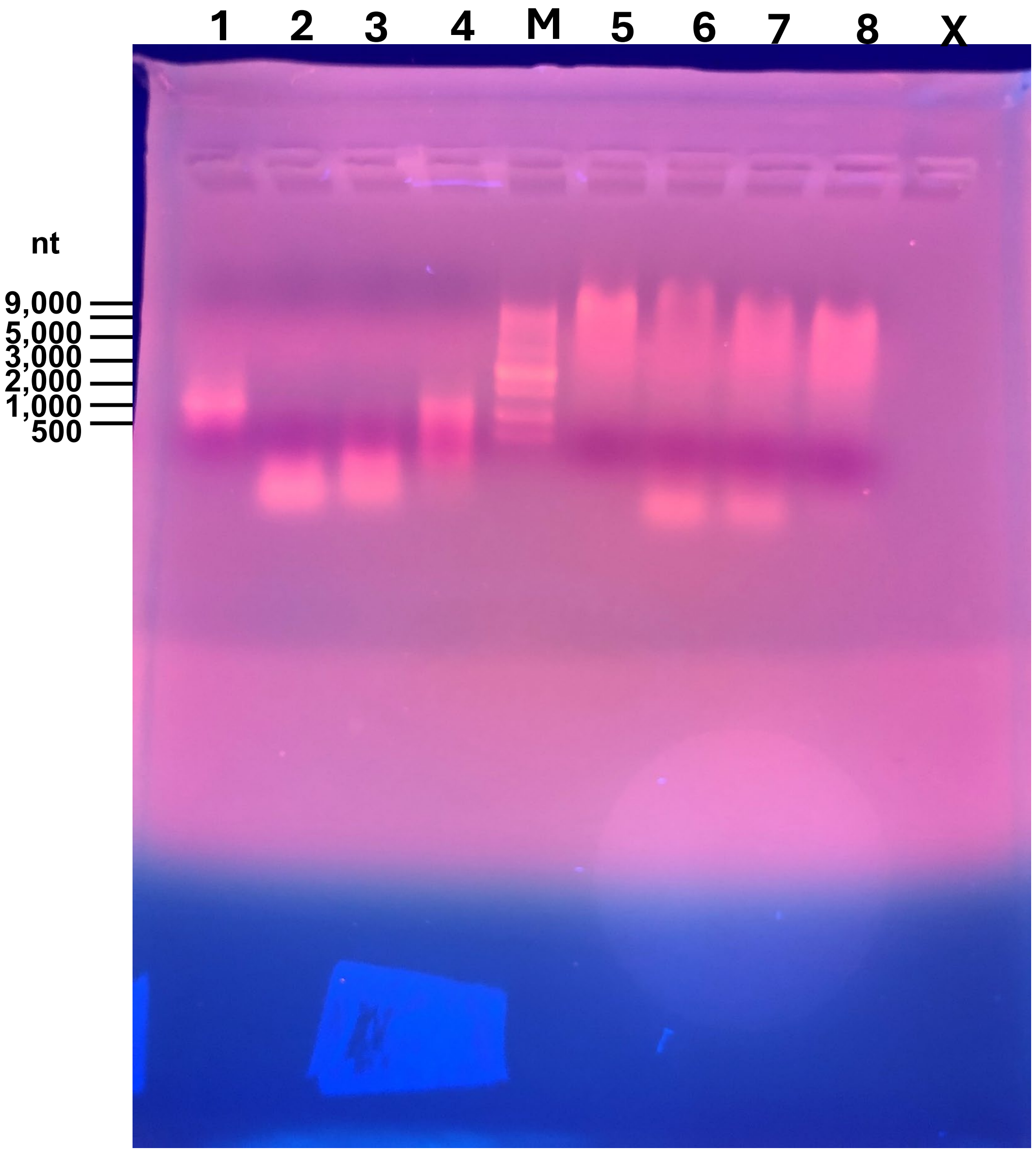

Figure 1 (B) In vitro transcribed N RNA was incubated with N at a 1:80 molar ratio to form N-RNA complexes (Lanes 5-8). As a control, free RNA was incubated under the same conditions without N (Lanes 1-4). Varying concentrations of RNase A (0.1 µg/mL, lanes 2 and 6; 0.01 µg/mL, lanes 3 and 7; 0.001 µg/mL, lanes 4 and 8) were added to the reactions for 30 minutes at 37 °C. The samples were resolved on a 1% non-denaturing agarose gel. Lane M contains an ssRNA ladder used to confirm the approximate size of the input RNA (~1200 nt).

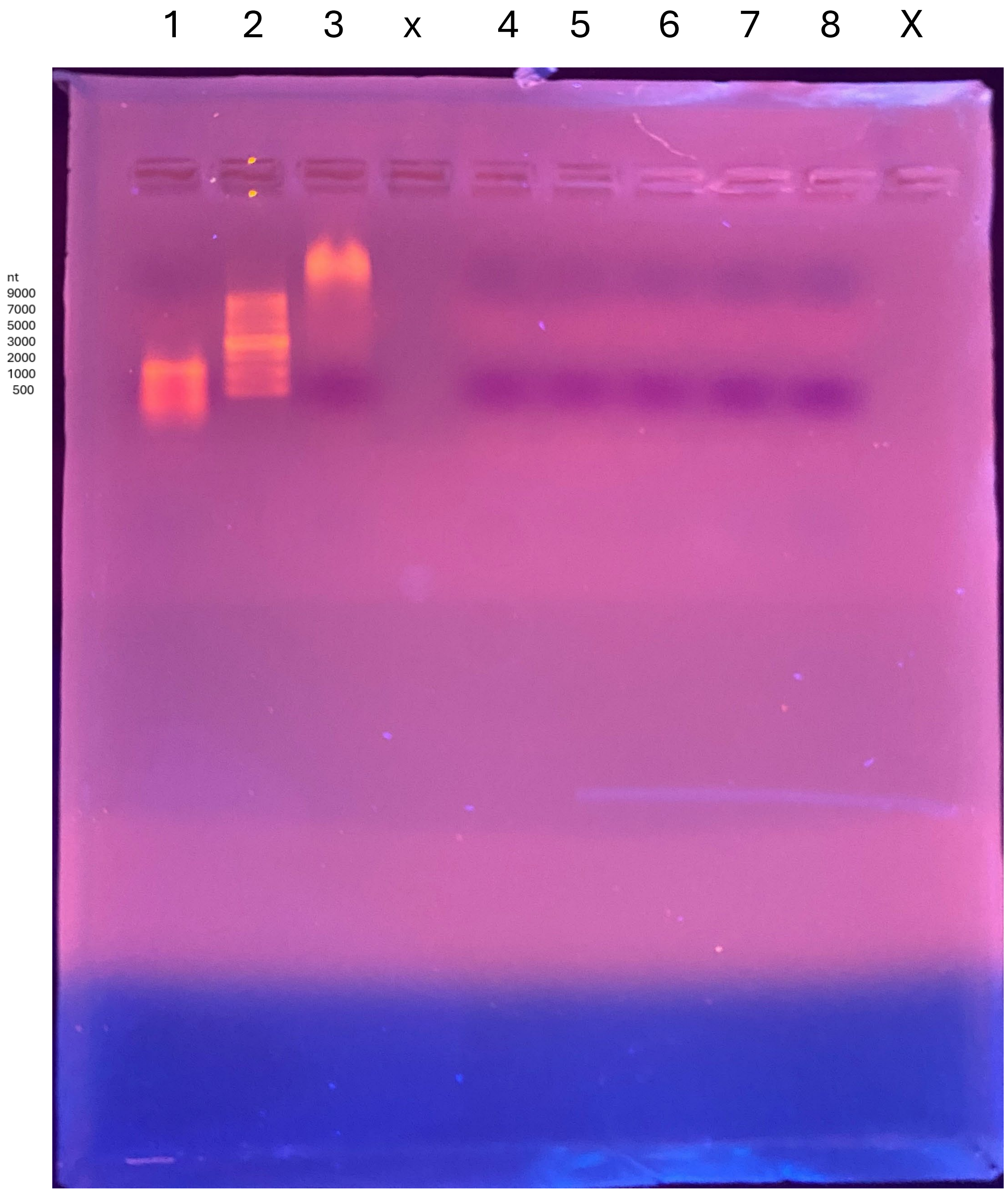

Supplementary Figure 1. Treatment with 10 μg/mL of RNAase A results in complete degradation of SARS-CoV-2 RNA in immune complexes. RNA-containing immune complexes were incubated with RNAase A (10 μg/mL) for 3 minutes (lane 4), 6 minutes (lane 5), 9 minutes (lane 6), 12 minutes (lane 7) or 15 minutes (lane 8). Lane 1: RNA alone; lane 2: RNA ladder (500-9,000 kb); lane 3: RNA-containing immune complexes untreated with RNAase A.

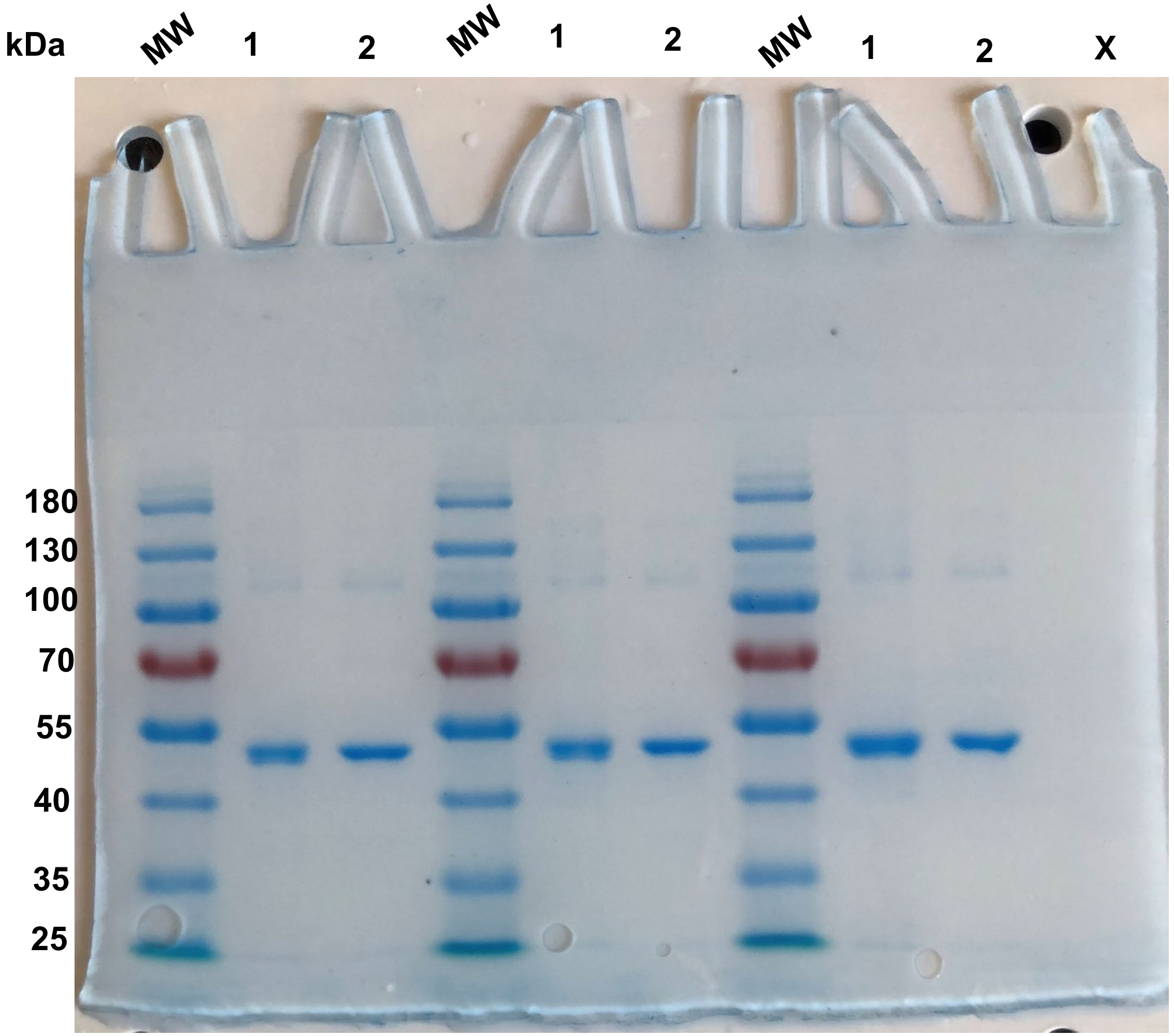

Supplementary figure 3 (A) Strep-Tactin purified CoV-2 N protein (lane 1, ~47 kDa) displays purity comparable to a commercial standard (lane 2) on a 10% non-reducing SDS-PAGE gel (3 µg loaded/lane).

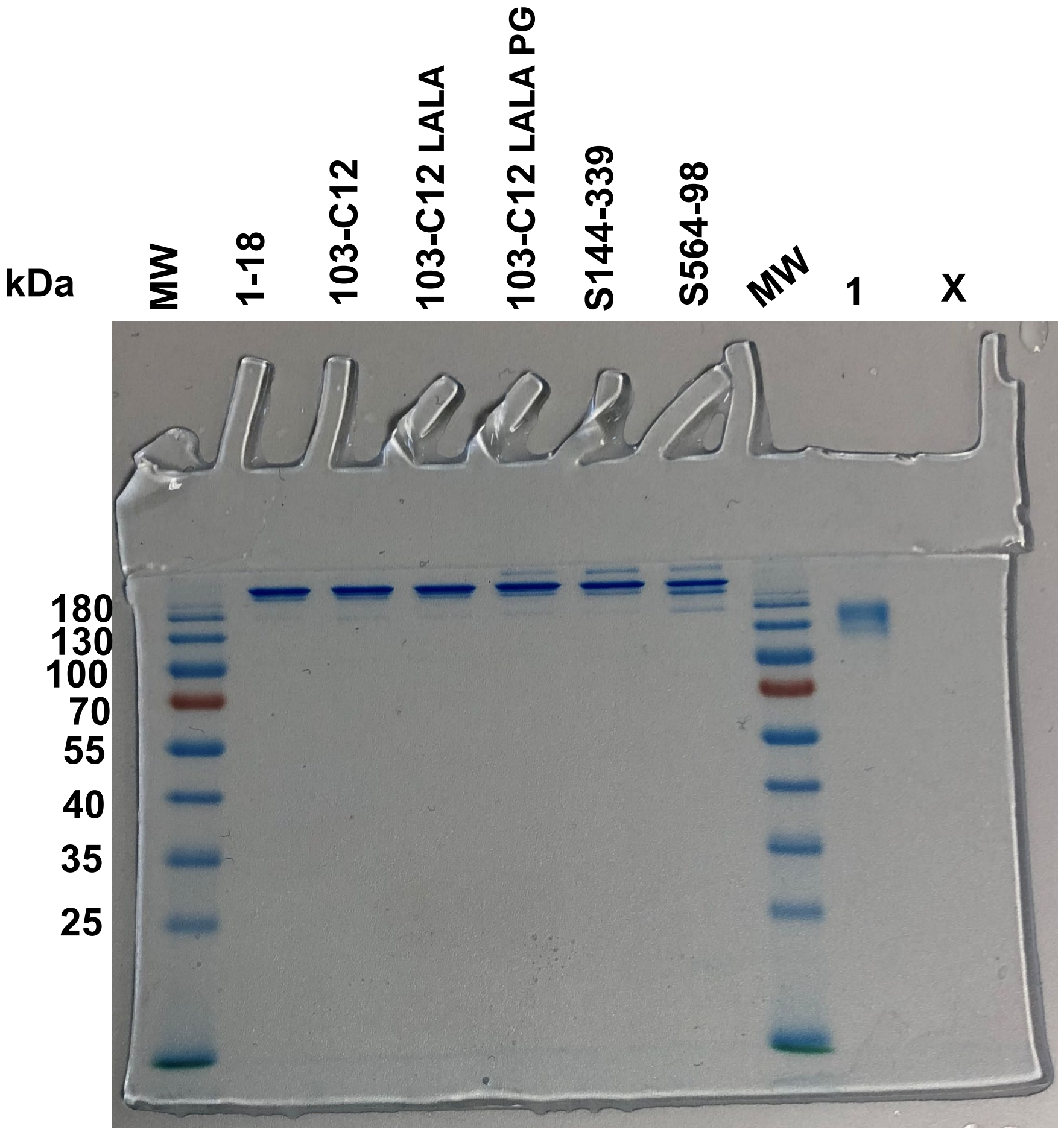

Supplementary figure 3 (B) Ni-NTA purified recombinant CoV-2 S protein (lane 1, ~130 kDa) runs as a single, highly pure band on a 12% SDS-PAGE gel (2 µg loaded).

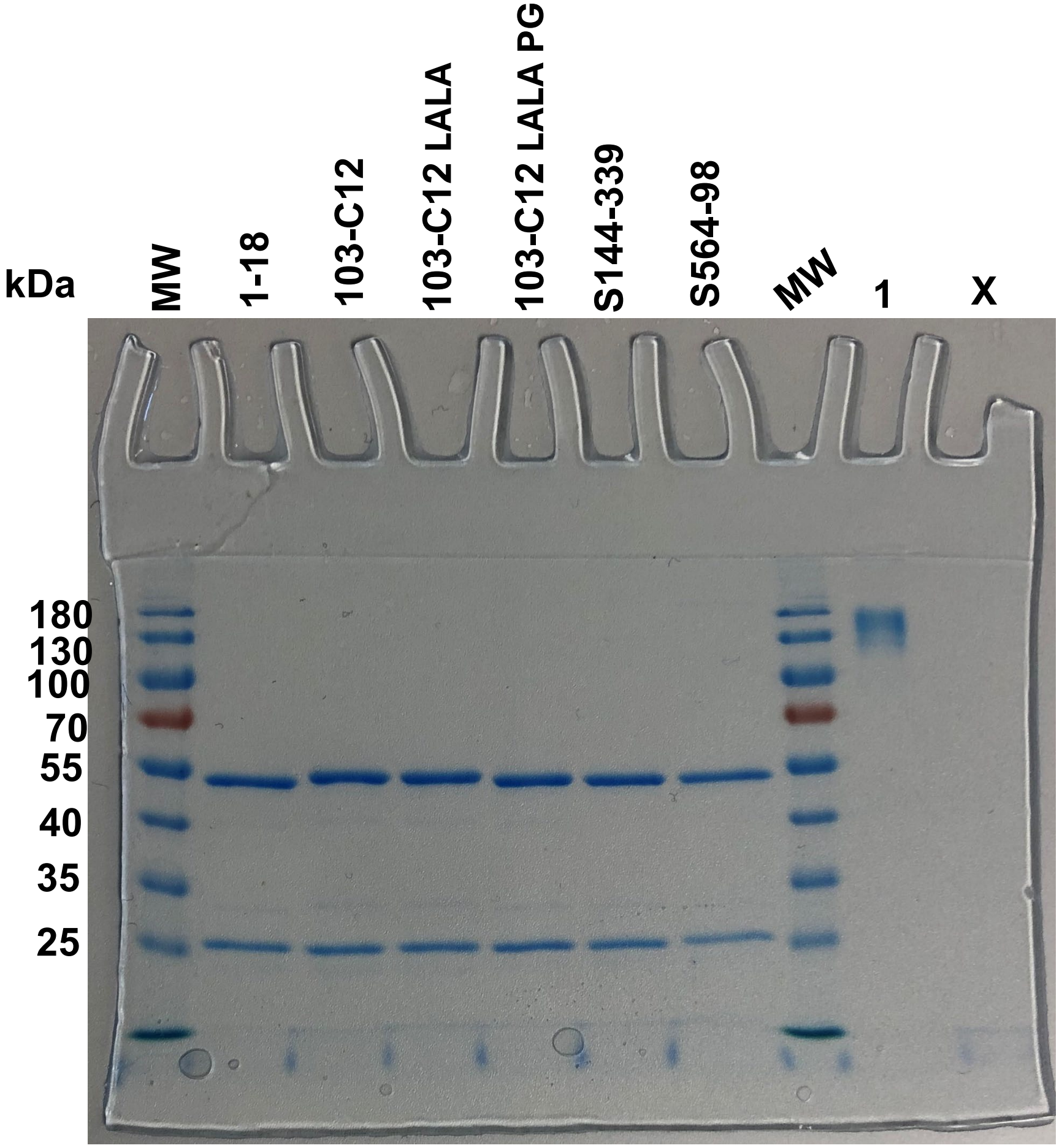

Supplementary figure 3 (**C**) Protein A-purified human mAbs targeting CoV-2 S and N proteins were analyzed under reducing conditions (1 µg loaded/lane) on a 10% SDS-PAGE gel.

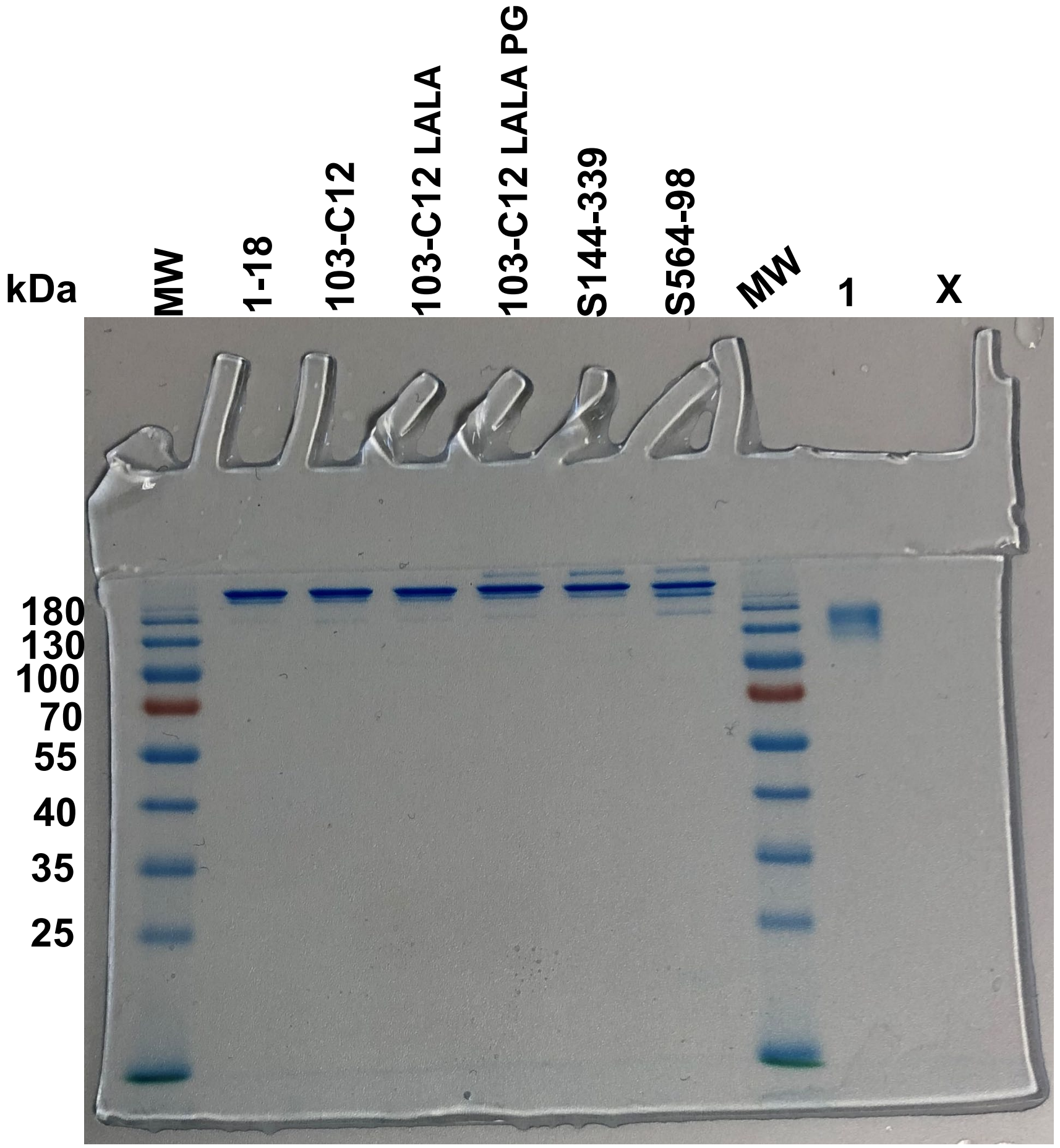

Supplementary figure 3 (**D**) Protein A-purified human mAbs targeting CoV-2 S and N proteins were analyzed under non-reducing conditions (1 µg loaded/lane) on a 10% SDS-PAGE gel.
